# Predictors of household food insecurity and relationship with obesity in First Nations communities in British Columbia, Manitoba, Alberta and Ontario

**DOI:** 10.1017/S1368980019004889

**Published:** 2021-04

**Authors:** Ashleigh Domingo, Jerry Spiegel, Martin Guhn, Hannah Wittman, Amy Ing, Tonio Sadik, Karen Fediuk, Constantine Tikhonov, Harold Schwartz, Hing Man Chan, Malek Batal

**Affiliations:** 1 Faculty of Applied Health Sciences, School of Public Health and Health Systems, University of Waterloo, Waterloo, ON N2L 3G1, Canada; 2 Faculty of Medicine, School of Population and Public Health, University of British Columbia, Vancouver, BC V6T 1Z3, Canada; 3 Faculty of Land and Food Systems, Centre for Sustainable Food Systems, University of British Columbia, Vancouver, BC V6T 1Z4, Canada; 4 Département de Nutrition, Faculté de Médecine, Université de Montréal, Montréal, QC H3T 1A8, Canada; 5 Environment, Assembly of First Nations, Ottawa, ON K1P 6L5, Canada; 6 School of Sociological and Anthropological Studies, University of Ottawa, Ottawa, ON K1N 6N5, Canada; 7 First Nations Food, Nutrition and Environment Study, University of Ottawa, Ottawa, ON, Canada; 8 Environmental Public Health Division, First Nations and Inuit Health Branch, Indigenous Services Canada, Ottawa, ON, Canada; 9 Department of Biology, University of Ottawa, Ottawa, ON K1N 6N5, Canada; 10 Centre de recherche en santé publique (CReSP), 7101 Avenue du Parc, Montreal, QC H3N 1X7, Canada

**Keywords:** First Nations communities, Indigenous health, Household food insecurity, Obesity

## Abstract

**Objective:**

To further understandings of household food insecurity in First Nations communities in Canada and its relationship with obesity.

**Design:**

Analysis of a cross-sectional dataset from the First Nations Food, Nutrition and Environment Study representative of First Nations communities south of the 60th parallel. Multivariate logistic regression was used to assess associations between food insecurity and sociodemographic factors, as well as the odds of obesity among food-insecure households adjusting for sociodemographic characteristics.

**Setting:**

Western and Central Canada.

**Participants:**

First Nations peoples aged ≥19 years.

**Results:**

Forty-six percent of First Nations households experienced food insecurity. Food insecurity was highest for respondents who received social assistance; had ≤10 years of education; were female; had children in the household; were 19–30 years old; resided in Alberta; and had no year-round road access into the community. Rates of obesity were highest for respondents residing in marginally food-insecure households (female 56·6 %; male 54·6 %). In gender-specific analyses, the odds of obesity were highest among marginally food-insecure households in comparison with food-secure households, for both female (OR 1·57) and male (OR 1·57) respondents, adjusting for sociodemographic variables. For males only, those in severely food-insecure (compared with food-secure) households had lower odds of obesity after adjusting for confounding (OR 0·56).

**Conclusions:**

The interrelated challenges of food insecurity and obesity in First Nations communities emphasise the need for Indigenous-led, culturally appropriate and food sovereign approaches to food security and nutrition in support of holistic wellness and prevention of chronic disease.

The pronounced health challenges faced by Indigenous peoples worldwide are deeply rooted in legacies of colonisation that have generated loss of land and erosion of cultural and social identity^(1–4)^. As a result, Indigenous peoples have been disproportionally affected by food insecurity – a challenge of uncertain or insufficient access to quality, healthy and personally and culturally appropriate/accepted food that persists in the Canadian context^(5–8)^.

In Canada, displacement from traditional territories, forced cultural assimilation, involuntary community relocation to reserves and environmental degradation have threatened Indigenous food systems and the resilience of communities to sustain a traditional diet and lifestyle^(9)^. The 1 673 785 individuals who self-identify as Indigenous in the 2016 Canada Census are a young and growing population, representing three distinct and diverse cultural peoples: First Nations (58·4 %), Inuit (3·9 %) and Métis (35·1 %)^(10)^. In 2016, among the 76·2 % of First Nations peoples with registered Indian status,^1^ approximately 44·2 % indicated primary residence being on reserve^2^ ^(4,10)^.

As widespread food insecurity among Indigenous households has accompanied transition away from traditional food sources, a nutritional burden has been placed on Indigenous peoples globally^(12–14)^. Alongside increased burden of chronic disease that has been observed such as type 2 diabetes, cancer and cardiovascular disease^(7,8,15–20)^, particular concern has been drawn to the increasing prevalence of obesity as a modifiable risk factor for diet-related chronic diseases that are reported to be higher among Indigenous peoples^(12–14,21–23)^. Understanding nutrition transition within the context of the dual and interrelated structural challenges of food insecurity and obesity confronting Indigenous peoples in Canada can support the adoption of culturally appropriate healthy nutritional policies, programmes and tools to be identified and informed by Indigenous peoples to reduce this nutritional burden and prevent chronic diseases^(12,21,23–26)^.

Over the past century, as First Nations communities have been subject to severe marginalising pressures including, but not limited to, the loss of traditional land, relationships, language and culture caused by colonisation^(27)^ and perpetuated by the residential school system^(28)^, they have experienced a rapid transition in diet and lifestyle, characterised by decreased physical activity and a shift towards consumption of energy-dense market-based food^(16,17,29,30)^. This nutrition transition has been identified to play a key role in the rising rates of obesity-related chronic diseases in these populations^(12,16,17,30–32)^, with this trend observed to be driven by a range of processes – first and foremost colonial policies and practices that enforced physical displacement from these lands, disrupting access to environments that enable engagement in traditional food activities^(2,3,27,28)^; a changing food environment from the introduction of various food sources and imported goods^(33)^; and increased engagement in market economy activity, leaving less time to engage in traditional food practices^(33)^.

In conjunction with this ongoing nutrition transition, the 2012 Canadian Community Health Survey (CCHS) observed disproportionately high rates of food insecurity among First Nations households (20 %) compared with non-Indigenous households in Canada (8 %)^(15)^. The CCHS, however, excludes First Nations households living on reserve, and therefore this prevalence of food insecurity is considered to underestimate the levels among all First Nations households^(34)^. In line with this, results from the First Nations Regional Health Survey Phase III, which includes Indigenous peoples living on-reserve and in northern communities, indicate 50·8 % of First Nations adults report their households as food-insecure^(35)^. While the nine food security questions in the First Nations Regional Health Survey are similar to the eighteen-item Household Food Security Survey Module in the CCHS, more than one individual within the same household could respond to the survey^(35)^, and this may therefore impact the estimated prevalence of food insecurity. A deepened understanding of food insecurity in First Nations communities and opportunities to address risks of chronic diseases, including its implications for holistic health and wellness, is therefore needed^(2,3,12,13,24–26)^.

Food insecurity has been associated with the inadequacy of food supplies in households, resulting in reduced quality or desirability of food consumed, including a low healthy eating index score tied to a decreased consumption of fruit and vegetables, dairy products and grains^(36)^, alongside attention to the importance of also considering ‘cultural food sovereignty’^(26)^. Reduced nutrient intake and poor dietary quality related to food insecurity have contributed to obesity with implications for risks of chronic diseases^(12,13,16,18,36,37)^.

The prevalence rates of obesity have also been found to be higher among First Nations people living on-reserve and in northern communities (34·8 %) as indicated in the First Nations Regional Health Survey 2008/2010^(35)^, as well as among First Nations individuals living off-reserve (31 %) in comparison with the total population in Canada (17 %)^(15)^. The relationship between food insecurity and excess weight is not well understood^(38)^. However, growing interest in food security as a determinant of health is expanding the emergence of literature on the positive association between food insecurity and obesity^(39–44)^. This relationship has been linked to eating patterns characterised by a high consumption of low-cost, energy-dense and nutrient poor foods^(16,42,44–46)^. This represents a dual interrelated concern whereby obesity is a challenge tied to an overabundance of energy-dense food, while the lack of affordable and accessible nutritious foods is an issue contributing to food insecurity. With First Nations communities observed to suffer disproportionally from both the burden of food security and of obesity^(23)^, understanding the patterns of food insecurity and ties with obesity, therefore, merits attention in order to identify culturally relevant strategies to address this dual public health challenge.

While a few studies have examined the association between food insecurity and obesity, existing research has yielded conflicting findings across women and men as well as across levels of food insecurity measured – and has primarily examined non-Indigenous populations in Canada^(40–42,44–46)^. To our knowledge, there has been no study that has investigated the association between food insecurity and obesity within an Indigenous context in Canada, in particular on-reserve First Nations communities. Further, as data from the CCHS excludes First Nations living on-reserve, diet-related health concerns associated with unique food security challenges faced by First Nations communities^(26)^ heighten the need for this nutritional and public health challenge to be assessed. This would support a better understanding of its differential effects within this population while not diminishing focus on the need to address the driving forces underlying food and nutrition challenges.

To deepen understandings of patterns of food insecurity and diet-related health concerns, our study examines the demographic and socioeconomic factors associated with household food insecurity in First Nations communities in four provinces of Canada, with attention to the relationship between household food insecurity and obesity. In the current study, the level of food insecurity reported from the 2008–2013 First Nations Food, Nutrition and Environment Study (FNFNES) was defined as a household’s inability to access food, including due to financial constraints^(47–51)^. The objectives of this article are to contribute to understandings of food insecurity regarding influences on its presence in First Nations households, as well as how this is associated with obesity. Given the challenges with understanding culturally appropriate indicators of food security, which includes consideration of access to traditional foods through practices such as hunting, gathering, fishing and trading, we wish to stimulate considerations about nutrition transition and the value of Indigenous-led approaches to health-promoting nutritional policies, programmes and tools to reduce the burden of food insecurity, obesity and chronic diseases.

## Methods

### Study

Data analyses were conducted using inputs from the FNFNES, a research initiative with a cross-sectional study design, regionally representative of First Nations communities south of the 60th parallel in Canada. FNFNES was supported by a resolution passed by the Assembly of First Nations in 2007 and was a collaborative partnership between the Assembly of First Nations, Indigenous Services Canada, the University of Northern British Columbia, the Université de Montréal and the University of Ottawa^(48–51)^. FNFNES was developed in response to growing concerns by First Nations communities regarding the presence of environmental contaminants in traditional food systems, and the need to document and address food-related health challenges across communities. This research was guided by the Tri-Council Policy Statement: Ethical Conduct for Research, including Chapter 9 of the Tri-Council Policy Statement, ‘Research Involving the First Nations, Inuit and Métis People of Canada^(52)^’. In addition, the First Nations principles of Ownership, Control, Access and Possession (OCAP^TM^)^(53)^ guided this undertaking to ensure ethical health research and appropriate engagement with First Nations communities. Engagement with First Nations leadership at the national, regional and community levels informed the FNFNES project design, including the survey questionnaire, study objectives, methodology and implementation, with funding support from the First Nations and Inuit Health Branch, Indigenous Services Canada. Ethical approval was obtained from the Ethics Research Board at Health Canada, the University of Northern British Columbia, the Université de Montréal, University of Ottawa and the University of British Columbia.

### Sampling frame

Using an ecological zone framework, a systematic multistage random sampling method was employed to select participants from communities from the provinces of British Columbia, Alberta, Manitoba and Ontario in Canada. This sampling strategy aimed to establish equal representation of communities from the Assembly of First Nations regions (in this case provinces) and ensure the likelihood of highly populated communities to be sampled. Within each community, questionnaire data were collected from a single adult (self-identified as of First Nations descent, age ≥19 years, living on-reserve) in each selected household. Within the selected households with more than one adult, an individual who self-identified as a First Nations person living on-reserve with the next birthday was invited to participate in the study.

Between 2008 and 2013 of the FNFNES’s ten-year project timeline (2008–2018), 5355 households from fifty-eight communities were randomly selected across British Columbia, Alberta, Manitoba and Ontario. Of the 5355 households sampled, a total of 3847 adults participated in household interviews, representing a participation rate of 74·5 %. Households were excluded from the survey if the respondent did not meet the inclusion criteria, was unable to participate due to health reasons (deafness, cognitive impairment) and if homes were vacant.

### Measures

Data utilised in the current study were collected from the social, health and lifestyle questionnaire and food security questionnaire administered to participating households by a trained community member. The social, health and lifestyle questionnaire in the FNFNES incorporates questions from the CCHS and previous work with Indigenous populations in Canada^(54)^ and collects information on general health status, sociodemographic characteristics and measured and self-reported height and weight. If accepted to be measured, weights were recorded using a Seca 803 digital scale with the participants lightly clothed. Height was measured using a measuring tape on shoeless participants on an even surface. BMI was calculated as weight/height (kg/m^2^) for 691 participants with both measured and self-reported height and weight, 922 participants with measured height and weight only, and 1749 participants with self-reported measures only. To account for a potential response bias among self-reported cases of height and weight, adjustments were made through the addition of estimated bias determined using a paired *t* test to estimate differences in means in BMI between measured and self-reported values. Adjustments were applied to self-reported BMI in Ontario and Alberta, but were not necessary for British Columbia and Manitoba. Participants were considered obese if their BMI was ≥30·0^(55)^.

The food security questionnaire used in the FNFNES is the income-related Household Food Security Survey Module. Household Food Security Survey Module, developed by the US Department of Agriculture, is internationally recognised as a measure of household food insecurity^(56)^ for comparative purposes and has been used in national surveys in Canada, such as the CCHS^(34)^. The food security questionnaire comprises eighteen questions to measure self-reports of household food insecurity due to financial constraints within a 12-month period prior to being surveyed. Ten of the eighteen questions measure adult experiences of food insecurity, and the remainder measure food insecurity among households with children under the age of 18. From the survey, the degree of household food insecurity was determined from a numerical scale based on the number of affirmed responses (‘yes’, ‘often’ or ‘sometimes true’, ‘almost every month’ or ‘some months, but not every month’). Household food security status was identified by categorising the severity of food insecurity from the food security numerical scale^(34,56)^. In the current study, household food security status was described using the following categories^(34)^: (i) food security (no affirmed response); (ii) marginal food insecurity (no more than one affirmed response on the adult food security scale or child food security scale); (iii) moderate food insecurity (2–5 affirmed responses on the adult food security scale, and 2–4 affirmed responses on the child food security scale); (iv) severe food insecurity (≥6 affirmed responses on the adult food security scale, and ≥5 affirmed responses on child food security scale).

### Statistical analysis

Prevalence estimates of household food security status were derived for marginal, moderate and severe food insecurity, as well as total household food (in)security. Prevalence estimates of obesity were also determined for female and male respondents living in food-secure households, as well as households experiencing marginal, moderate and severe food insecurity.

Multivariate logistic regression modelling was carried out in the current study to examine: (i) sociodemographic predictors of household food insecurity, and (ii) the relationship between household food insecurity and obesity in First Nations communities in British Columbia, Manitoba, Alberta and Ontario. In the first model, sociodemographic predictors of household food insecurity were examined through multivariate logistic regression modelling using the backwards-stepwise selection method. The outcome variable of interest, household food insecurity, was dichotomised as food-secure *v*. food-insecure (combining marginal, moderate and severe subcategories). Sociodemographic predictors identified in a review of the literature (Table 1) were entered into the model, and the least significant predictor was removed in a stepwise process using a preliminary significance level of *α* <0·1. The fit of the model was assessed using the Akaike Information Criterion and Hosmer and Lemeshow goodness-of-fit test. Collinearity was assessed and variables identified to be strongly associated with each other were removed from the model. Two-way interactions were examined among significant predictors contained in the model; however, significant interactions that contributed meaningfully to the model were not found. Predictors retained in the final model contributed to model fit and met statistical significance at *P* < 0·05 and based on the Type III Sum of Squares.

**Table 1:** Variables explored as potential predictors of food insecurity among First Nations respondents*

	Predictor	Description	Type
Individual level	Sex	Whether the respondent of the household was female or male	Categorical
	Age group	Age of the respondent categorised as 19–30, 31–50, 51–70, 71+	Categorical
	Years of education	Number of years of education of the respondent	Continuous
Household level	Income source†	Main source of income categorised as workers’ compensation/employment insurance; wages; social assistance; pension; other	Categorical
	Region	Province where the respondent resides, categorised as British Columbia (BC), Manitoba (MB), Ontario (ON), Alberta (AB)	Categorical
	Presence of children in the household	Whether the respondent indicated yes or no to children being present in the household	Categorical
	Total people in the household	Number of people in the household	Continuous
	Climate‡	Whether climate change has impacted the availability of traditional food in the household	Categorical
	Contaminants§	Whether environmental contaminants (pollution, pesticides, industrial effluents) prevented the household from using more traditional food	Categorical
Community level‖	Community road access	Whether the community is accessible year-round, by plane only or by winter road	Categorical
	Food basket costs	Cost and affordability of healthy eating within each community	Continuous
	Distance to service centre (km)	Distance to service centre from each community	Continuous

FNFNES, First Nations Food, Nutrition and Environment Study; SHL, social, health and lifestyle.

* FNFNES. Data are unweighted.

† Measure of income as captured by the SHL questionnaire in FNFNES was the main source of information. The questionnaire does not capture annual wages and salaries among household respondents.

‡ Based on question 9a and 9b of the SHL questionnaire in FNFNES.

§ This variable was obtained from question 8b of the SHL questionnaire in FNFNES.

‖ Food basket costs were measured based on sixty-seven basic food items using Health Canada’s 2008 National Nutritious Food Basket Tool. Distance to service centre was measured from road access to a service centre (access to banks, suppliers and government services)^(47–49,57)^.

In the second model, the relationship between household food insecurity and obesity was examined with bivariate and multivariate logistic regression to assess the odds of obesity for individuals living in food-insecure households. The outcome variable, BMI, was dichotomised to assess the presence or absence of obesity in First Nations households by food insecurity status. The variable, household food insecurity status, was the main effect of interest and was classified into marginal food insecurity, moderate food insecurity, severe food insecurity and food security. Bivariate analyses were carried out to assess differences in the association between obesity and the primary explanatory variable, household food insecurity, with each sociodemographic variable of interest. Statistical effect sizes for household food insecurity were estimated for female and male respondents separately using the three levels of food insecurity. In the multivariate model, the odds of obesity among food-insecure households was determined adjusting for the variables age group, region, main source of income and years of education (Table 1). Unadjusted and adjusted OR were calculated, and 95 % CI for OR were obtained. Although no significant interactions were found in the multivariate model, gender-specific analyses were conducted to account for differences in dietary quality between females and males. All statistical analyses were conducted using the Statistical Analysis Software (SAS), version 9.4 (SAS Institute), and observations with missing values were excluded.

## Results

Table 2 provides a description of demographic and socioeconomic characteristics of the 3681 participants who responded to the household food security questionnaire (2370 women and 1311 men). Food security status responses of participants who answered ‘don’t know/refused’ to at least one of the first three food security questions were excluded and treated as missing values. Participants’ age ranged from 19 to 96 years, and the average age of female and male respondents was 44 and 46 years, respectively. Almost half (46·4 %) of these respondents reported some level of household food insecurity, with 9·5 % experiencing marginal food insecurity, indicative of concerns regarding adequate and secure access to food; 27·9 % experiencing moderate food insecurity, demonstrating experiences with compromises in the quality and/or quantity of food; and 8·9 % experiencing severe food insecurity, reporting a disruption of eating patterns and reduced food intake. For households where the respondent was female, 10·6 % experienced marginal food insecurity, 29·6 % were moderately food-insecure and 8·7 % demonstrated severe food insecurity. In households where the respondent identified as male, 7·7 % experienced marginal food insecurity, while 24·9 % were categorised as moderately food-insecure and 9·3 % reported severe food insecurity.

**Table 2:** Distribution of study participants by demographic and socioeconomic characteristics*

Characteristics	Overall		Women		Men	
	*n*	%†	*n*	%‡	*n*	%‡
Completed social, health and lifestyle questionnaire	3847	100	2465	64·1	1382	35·9
Age group	3826	100	2451	[100] 64·1	1375	[100] 35·9
19–30	782	20·4	527	21·5	255	18·6
31–50	1767	46·2	1163	47·5	604	43·9
51–70	1059	27·7	627	25·6	432	31·4
71+	218	5·7	134	5·47	84	6·11
Region	3847	100	2465	[100] 64·1	1382	[100] 35·9
Alberta	609	15·8	387	15·7	222	16·1
British Columbia	1103	28·7	705	28·6	398	28·8
Manitoba	706	18·4	477	19·4	229	16·6
Ontario	1429	37·2	896	36·3	533	38·6
Incomes source	3806	100	2439	[100] 64·1	1367	[100] 35·9
Wages	1916	50·3	1269	52·0	647	47·3
Social assistance	1053	27·7	650	26·7	403	30·0
Pension	507	13·3	316	13·0	191	14·0
Workers’ compensation/employment insurance	230	6·0	134	5·5	96	7·0
Other	100	2·6	70	2·9	30	2·2
Average years of education						
Mean	10·7		10·9		10·3	
95 % CI	10·6, 10·8		10·8, 11·1		10·1, 10·4	
Presence of children in the household	3847	100	2465	[100] 64·1	1382	[100] 35·9
Yes	2277	59·2	1636	66·4	641	46·4
No	1570	40·8	829	33·6	741	53·6
Community road access	3847	100	2465	[100] 64·1	1382	[100] 35·9
Year-round	3131	81·4	2003	81·3	1128	81·6
Winter road only	469	12·2	289	11·7	180	13·0
Fly in	247	6·4	173	7·0	74	5·3
BMI classification	3364	100	2048	[100] 60·9	1316	[100] 39·1
Obesity (≥30·0)	1548	46·0	1002	48·9	546	41·5
Overweight (25·0–29·9)	1133	33·7	626	30·6	507	38·5
Normal weight (18·5–24·9)	646	19·2	396	19·3	250	19·0
Underweight <18·5	37	1·1	24	1·2	13	1·0
Completed household food security survey	3681	100	2370	[100] 64·4	1311	[100] 35·6
Household food insecure	1707	46·4	1157	48·8	550	42·0
Marginal	351	9·5	250	21·6	101	18·4
Moderate	1028	27·9	701	60·6	327	59·6
Severe	328	8·9	206	17·8	122	22·2
Household food secure	1974	53·6	1213	51·2	761	58·1

* First Nations Food, Nutrition and Environment Study. Data are unweighted. Totals may vary due to the exclusion of missing values for participants of the study. Percentages may not add up to 100 % due to rounding.

† % distributions for categories and sub-categories.

‡ % distributions for each subcategory category by gender [gender distribution of category total].

The prevalence of food insecurity varied across demographic and socioeconomic characteristics of the household (Table 3). In multivariate analyses, the rates of food insecurity were highest among surveyed household respondents who were female (49·1 %), between the ages of 19 and 30 (55·4 %), from Alberta (54 %), in households with children (53·4 %) and receiving social assistance as a main source of income (66·3 %) (Table 4). Respondents from households that were food-insecure had, on average, 10 years of education, while respondents from food-secure households had an average of 11 years of education. Among households with children, 32·5 % experienced moderate food insecurity, 11·1 % reported marginal food insecurity and 8·7 % were severely food-insecure. In comparison, 21·4 % of households without children experienced moderate food insecurity, while 7·6 % indicated being marginally food-insecure and 9·2 % were severely food-insecure. When compared with food-secure households with children (<18 years of age), food-insecure households with children had a greater average number of individuals residing in the household (5·2 compared with 4·8) and a larger ratio of persons under 15 years to persons ≥15 years of age in the household (1·1 compared with 0·97).^3^ Additionally, higher rates were found among First Nations households in communities with limited road access (63·3 % in the five communities accessible by plane only, and 63·2 % in the seven communities accessible by winter road only). Table 4 presents the adjusted OR for food insecurity among First Nations households for each demographic and socioeconomic variable in the final multivariate logistic regression model of significant predictors of food insecurity. No statistically significant first-order interactions were found in the multivariate analysis.

**Table 3:** Prevalence and bivariate analysis of predictors of food insecurity*

Predictor	*n*	Food-secure (%)		Food-insecure (%)		*P* †
		*n*	%	*n*	%	
Sex	3681	1974	53·6	1707	46·4	<0·001
Female	2370	1213	51·2	1157	48·8	
Male	1311	761	58·1	550	42·0	
Age group	3663	1966	53·7	1697	46·3	<0·001
19–30	747	350	46·9	397	53·2	
31–50	1687	848	50·3	839	49·7	
51–70	1023	625	61·1	398	38·9	
71+	206	143	69·4	63	30·6	
Region	3681	1974	53·6	1707	46·4	<0·001
Alberta	594	274	46·1	320	53·9	
British Columbia	1065	564	53·0	501	47·0	
Manitoba	646	334	51·7	312	48·3	
Ontario	1376	802	58·3	574	41·7	
Income source	3642	1950	53·5	1692	46·5	<0·001
Wages	1858	1127	60·7	731	39·3	
Social assistance	980	332	33·9	648	66·1	
Pension	487	319	65·5	168	34·5	
Workers’ compensation/employment insurance	222	119	53·6	103	46·4	
Other	95	53	55·8	42	44·2	
Years of education	3608					<0·001
Mean		11·0		10·4		
95 % CI		10·9, 11·2		10·3, 10·6		
Presence of children in the household	3681	1974	53·6	1707	46·4	<0·001
Yes	2163	1030	47·7	1133	52·3	
No	1518	944	62·1	574	37·8	
Climate	2616	1410	53·9	1206	46·1	0·176
Yes	245	122	49·8	123	50·2	
No	2371	1288	54·3	1083	45·7	
Contaminants	3311	1759	53·1	1552	46·9	0·669
Yes	46	23	50·0	23	50·0	
No	3265	1736	53·2	1529	46·8	
Community road access	3681	1974	53·6	1707	46·4	<0·001
Year-round	3023	1732	57·3	1291	42·7	
Plane only	234	86	36·8	148	63·3	
Winter road only	424	156	36·8	268	63·2	
Food basket costs	2571					<0·001
Mean		214·3		237·8		
95 % CI		211·4, 217·3		233·7, 242·0		
Distance to service centre (km)	3681					<0·001
Mean		104·6		150·9		
95 % CI		98·6, 110·6		142·7, 159·0		

* First Nations Food, Nutrition and Environment Study. Data are unweighted.

† *P*-values correspond to a bivariate analysis of food security status and predictor and were determined from either *χ*^2^ test or *t* test.

**Table 4:** Prevalence of food insecurity by sociodemographic predictor variables and adjusted OR for household food insecurity (*n* 3224)*

Predictor variable	*n*	Food-insecure (%)		Adjusted analysis			
		*n*	%	OR	95 % Wald CI		*P* †
Sex	3224	1504	46·7				
Male	1146	483	42·2	Ref.	–		
Female	2078	1021	49·1	1·31	1·11	1·53	<0·001
Age group							
71+	187	54	28·9	Ref.	–		
19–30	639	354	55·4	2·23	1·39	3·57	0·001
31–50	1497	757	50·6	2·19	1·40	3·45	0·001
51–70	901	339	37·6	1·54	1·01	2·33	0·043
Region							
Ontario	1342	556	41·4	Ref.	–		
Alberta	583	315	54·0	1·62	1·28	2·06	<0·001
British Columbia	677	338	49·9	1·77	1·43	2·20	<0·001
Manitoba	622	295	47·4	0·98	0·77	1·25	0·888
Income source							
Wages	1638	654	39·9	Ref.	–		
Workers’ compensation/employment insurance	184	90	48·9	1·79	1·30	2·48	0·0004
Social assistance	869	576	66·3	3·00	2·48	3·63	<0·001
Pension	439	142	32·4	1·26	0·93	1·72	0·141
Other	94	42	44·7	1·20	0·78	1·86	0·406
Years of education	3224			0·97	0·94	0·99	0·017
Mean		10·4					
95 % CI		10·3, 10·6					
Presence of children in the household							
No	1337	496	37·1	Ref.	–		
Yes	1887	1008	53·4	1·52	1·28	1·80	<0·001
Community road access							
Year-round	2633	1124	42·7	Ref.	–		
Plane only	407	125	67·9	2·61	1·84	3·70	<0·001
Winter road only	184	255	62·7	2·83	2·16	3·71	<0·001

* First Nations Food, Nutrition and Environment Study. Data are unweighted.

† *P*-values correspond to a multivariate logistic regression analysis of food security status and predictor variables. *R*^2^ = 17 %. The analyses excluded participants with missing values for all predictor variables accounted for in the model. The variables excluded in the backward-stepwise selection method due to a lack of statistical significance and power include climate, contaminants and food basket costs.

The prevalence rates of obesity were also high among the 3364 participants with calculated BMI in the sample, with minor differences reported between food-secure and food-insecure households. For both female and male respondents, the prevalence rates of obesity were highest in marginally food-insecure households and lowest for severe food-insecure households (Table 5). Female respondents living in marginally food-insecure households had an average BMI of 31·8 kg/m^2^. The average BMI for female respondents living in moderate food-insecure households was 30·4 and 29·3 kg/m^2^ for females residing in severely food-insecure households. For male respondents residing in marginally food-insecure households, the average BMI was 30·2 kg/m^2^, while male respondents living in moderate and severe food-insecure households had an average BMI of 28·9 and 28·2 kg/m^2^, respectively.

**Table 5:** Prevalence of obesity by food security status for women and men*

	Women (%)		Men (%)	
	*N*	%	*n*	%
Food-secure	488	48·0	323	44·6
Food-insecure	473	49·5	199	37·8
Marginally food-insecure	116	56·6	53	54·6
Moderately food-insecure	286	49·1	111	35·5
Severely food-insecure	71	42·3	35	29·9

* First Nations Food, Nutrition and Environment Study. Data are unweighted. Percentages may not add up to 100 % due to an exclusion of participants with a BMI classification of overweight (25.0–29.9), normal weight (18·5–24·9) or underweight (<18·5).

In both bivariate and multivariate logistic regression gender-specific analyses, the odds of obesity were highest among marginally food-insecure households in comparison with food-secure households. Table 6 shows the gender-specific unadjusted and adjusted OR for obesity among individuals living in marginally, moderately and severely food-insecure households compared with individuals living in food-secure households. After adjustments were made for age, income, years of education and region, higher odds of obesity among marginally food-insecure households were observed for both male and female respondents. For male respondents only, lower odds of obesity among severely food-insecure households were found after adjusting for confounding variables.

**Table 6:** Logistic regression analysis of obesity associated with three different levels of household food insecurity†

	Women (*n* 1905)				Men (*n* 1221)			
	Unadjusted OR		Adjusted OR‡		Unadjusted OR		Adjusted OR‡	
	OR	95 % CI	OR	95 % CI	OR	95 % CI	OR	95 % CI
Food-secure§	–		–		–		–	
Marginally food-insecure	1·46*	1·08, 1·98	1·57**	1·15, 2·15	1·51	0·98, 2·32	1·57*	1·01, 2·43
Moderately food-insecure	1·04	0·84, 1·28	1·12	0·90, 1·39	0·69**	0·52, 0·91	0·77	0·58, 1·03
Severely food-insecure	0·8	0·57, 1·11	0·89	0·63, 1·27	0·52**	0·34, 0·79	0·56*	0·36, 0·87

*P < 0·05; **P < 0·01; significantly different from food security.

† First Nations, Food Nutrition and Environment Study. Data are unweighted.

‡ OR from a multivariate logistic regression adjusting for age, income, years of education and region. The analyses excluded participants with missing values for all variables accounted for in the model.

§ Reference category.

## Discussion

The FNFNES provided the opportunity to examine the patterns of food insecurity, as well as its associations with obesity in First Nations communities, south of the 60th parallel, in the Canadian provinces of British Columbia, Alberta, Manitoba and Ontario. As one of the first published examinations of this dual interrelated challenge among First Nations households, our study contributes to the scholarship on nutrition transition and emerging literature focusing on food insecurity and obesity as priority areas for action.

A very high prevalence of food insecurity (46·4 %) was observed among First Nations households surveyed in First Nations communities of Canada. This reveals a particular challenge to ensuring food security compared with the considerably lower 2012 CCHS levels of food insecurity among the general Canadian population that have attracted considerable concern (British Columbia 12·7 %; Alberta 11·5 %; Ontario 11·7 %; Manitoba 12·1 %)^(34)^. Differences in the levels of food insecurity observed confirm how the omission of First Nations living on-reserve in the CCHS could underestimate the degree of food insecurity being experienced across Canada, as well as draw attention to the extreme injustice presented by the disproportionately higher rates for First Nations living on-reserve. This has implications for identifying Indigenous-led health promotion and prevention strategies and ensuring that approaches taken are culturally safe and reflective of Indigenous priorities.

The predictors of food insecurity that are associated with highest odds of food insecurity within First Nations households – households within communities without year-round road access, respondents who are female, receiving social assistance, presence of children in the household, years of education ≤10, younger age (< 50) and residing in British Columbia or Alberta – highlight points for interventions to be considered. These sociodemographic characteristics were found to be significantly associated with higher odds of household food insecurity, consistent with studies examining the determinants of food insecurity in Indigenous households in other Canadian settings^(58–61)^.

The interrelated challenge of food insecurity and obesity observed in our study has its roots in land dispossession and the ongoing impacts of colonisation that have undermined Indigenous peoples’ connection to the land and its resources^(9)^. Although we did not directly probe these drivers, the differences we observed among the predictors of First Nations household food insecurity can be best understood within the context of delocolonisation of food systems and the underlying stresses to traditional, historically sustaining Indigenous food systems. These stressors to Indigenous food systems are related to land dispossession, forced cultural assimilation through residential schools, and community relocation, which have been examined in other published work in Canada^(9,27,28)^. For many Indigenous communities, the natural environment as a context for accessing food, water and livelihood represents an essential pathway to food security^(5,26)^. Through gathering, harvesting and sharing of local plant or animal resources, the holistic health and wellness of Indigenous peoples has long been sustained by their food systems and deep relations with the environment^(3,26)^.

As experiences of food insecurity differ between women and men, we recognise our inability to identify specific details, such as individual’s marital status or whether female respondents were responsible for food preparation, as a potential limitation of our study’s questionnaire design. Further, gender inequalities faced by Indigenous women as a consequence of reduced access to resources, such as land, education and agricultural inputs, have been previously exposed as structural determinants of food insecurity^(9,62)^. Studies have also found that women compromise their own food security by altering their eating patterns to ensure other members within the household are food-secure^(63–65)^. For example, when food is limited in the household, women have been described to skip meals or delay eating in order to prevent hunger among children in the household^(55,56)^. While the high prevalence of food insecurity among female respondents may point towards efforts taken by them to prevent food insecurity among children, findings consistently show that households with children experience greater food insecurity compared with households without children^(34,63)^.

In Canada, low income levels have been identified as a primary barrier to healthy eating or obtaining food that meets individual’s preferences and needs^(66,67)^. In the FNFNES survey, from which the current study has drawn major inputs, precise income level was not measured as an indicator of low socioeconomic status, although household crowding, receiving social support and education level were considered^(36)^. Food insecurity in households with children is most prevalent among families led by a single parent and households depending on social assistance as a source of income^(67,68)^. The presence of more family members has placed an increased burden on household resources with implications on food insecurity^(67,69)^. With regard to households with children, food-insecure households had, on average, more individuals living within the household compared with those that were food-secure. Further, food-insecure households had a greater number of individuals under the age of 15 (ineligible to participate in the labour market) who may have limited capacity to contribute to their household’s overall income. An association between food insecurity and income levels also indicates that households receiving social assistance or dependent on employment insurance/workers’ compensation are at a higher risk as these sources of income may not be sufficient to ensure food security^(67,70)^.

While the current study was not designed to conduct a provincial comparative analysis of food insecurity, households within the province of British Columbia and Alberta were at higher odds of food insecurity compared with those in the province of Ontario. Provincial variations observed in food insecurity are likely to be tied to differences in the average cost of food in those provinces^(48–50,71)^ and differences in provincial social assistance policies and programmes^(72)^. Additional factors may be linked to the number of participating communities located in rural, remote or isolated regions within each province, as households residing in communities accessible by winter road or plane only had higher rates of food insecurity compared with households in communities with year-round road access.

We notably examined the dual interrelated challenges of food insecurity and obesity in the current study. While a non-linear association observed between household food insecurity and obesity derives from a cross-sectional design and, therefore, does not infer causality, the public health challenge that this exposes to exist calls for identifying culturally relevant approaches to reduce chronic disease risk factors among First Nations communities.

In our study, higher odds of obesity were found for both female and male respondents living in marginally food-insecure households compared with food-secure households. In comparison to women living in food-secure households, women living in marginally food-insecure households had higher odds of obesity, an association similar to that observed among men. However, the odds of obesity for men living in severe food-insecure households were lower in comparison to men living in food-secure households. While an association between living in severe food-insecure households and obesity was observed among women, this was not statistically significant. These results are consistent with findings of higher levels of overweight or obesity at low or intermediate levels of food insecurity and lower BMI observed at severe levels of food insecurity compared with food-secure households in non-Indigenous populations^(40–43)^.

A rapid change in diet and lifestyle, characterised by a forced shift from traditional food use towards consumption of energy-dense market-based foods, has placed a high burden of chronic diseases among Indigenous peoples globally^(13,33,70,73)^. This observed transition is characterised by an involuntary decrease in the use of traditional foods over time across Indigenous populations and particularly among younger individuals^(13,31)^. With an extensive documentation of a steep growth in the prevalence of obesity over time^(23)^, nutrition transition and links to chronic diseases are indicative of a forced transformation in diet and lifestyle observed in a context where options have been influenced by processes favouring reliance on processed foods^(2,3,16,17,30–33,74–76)^.

Consistent with prior observations of similar associations linking higher obesity with lower levels of food security, these studies often point to the reduced quality of purchased food when concerns about food access are present^(40,44)^. Energy-dense, nutrient-poor food is often made available at low cost and is usually consumed by households experiencing financial constraints^(46)^. For this reason, risks of obesity from diets composed of affordable, energy-dense foods are a concern among food-insecure households^(46)^, particularly in households with ‘marginal food insecurity’ whose food choice is limited due to a lack of money. In a study examining community perspectives on the relationship between food insecurity and obesity, First Nations and Métis parents perceived low income as an underlying factor of food insecurity, and reliance on energy-dense food as the most affordable coping option^(72)^. Similarly, changes in dietary practices observed among Inuit communities, especially those receiving income support, have also revealed their vulnerability to purchasing processed foods^(72)^.

Contrastingly, when households experience compromises in the quantity of food or reduced food intake due to severely food insecure levels, differential impacts on individual body weight may be observed. The lower odds of obesity observed in the current study among severe food-insecure households may be tied to a decrease in energy intake due to underconsumption and disrupted eating patterns, contributing to weight loss over time. Decreased consumption and reduced energy intake have been shown to lower obesity at higher levels of food insecurity^(40)^.

Given the persistence of structural determinants driving the association of obesity with food insecurity, it is critical that solutions be explored not in individualised behavioural approaches often characterised as ‘shaming’ but rather on modifications to the circumstances undermining food security itself – and in a culturally appropriate manner^(77)^. Such approaches could strengthen local food systems and reduce the burden of food insecurity and obesity that is highly prevalent in the First Nations communities.

The results of the current study substantiate the need for further investigations considering dietary intake and food supply when examining the association between food insecurity and obesity within First Nations communities. Until further work is undertaken, the current results should be interpreted with caution. However, given the health inequities already faced by this population, the potential consequences of both food insecurity and obesity on individual’s health and community wellness may risk furthering health challenges. The high and disproportionate burden of food insecurity and obesity among First Nations communities, therefore, represents priority areas for action. A consideration for food sovereign approaches in identifying interventions to strengthen local food systems can present opportunities to improve equitable access to healthy, affordable food with attention given to cultural appropriateness. This is necessary as efforts to mitigate food insecurity without concern for nutritional quality and access to culturally relevant food may limit the impact of prevention and health-promoting strategies aimed at addressing food insecurity and obesity.

Strategies targeted at improving the availability and access to affordable, culturally appropriate, healthy foods are essential for promoting sustainable and community-driven actions to ensure food security among First Nations households in remote or isolated communities. Indigenous-led food sovereign approaches aimed at protecting traditional food practices and improving access to culturally appropriate, healthy and affordable foods can present opportunities to ensure long-term food security against ongoing colonial pressures^(78)^. Engagement with First Nations, Inuit and Métis communities, the government and key stakeholders working in the areas of food, nutrition and chronic diseases could inform the development of sustainable and multifaceted approaches in support of Indigenous-led action on food insecurity.

The current study contributes to improving the understanding of factors underlying food insecurity and obesity in First Nations communities, thus facilitating the needed discussion on the value of Indigenous-led food sovereign approaches to enhance food security, including access to culturally appropriate, healthy and affordable food. There are, however, limitations to the current study. While the data utilised from FNFNES are regionally representative of First Nations communities in Canada, the cross-sectional design of the study presents limitations with regard to drawing inferences on a cause-and-effect relationship of the association between food insecurity and obesity. Additional socioeconomic and demographic characteristics related to the prevalence of household food insecurity were not measured by the FNFNES survey, such as the precise income level, marital status, family medical history and disordered eating patterns. Future studies led and informed by Indigenous partners and communities that assess community-based interventions to improve food security and nutrition are needed to identify culturally appropriate responses.

## Footnotes

1 Registered Indian refers to a person who is registered under the Canadian Indian Act that was initially passed in 1876^(11)^.

2 A reserve is Crown land designated by the Canadian federal government for the use and benefit by First Nations peoples^(4)^. The term community is used throughout this article to refer to a reserve.

3 A ratio of <15 years to ≥15 years of age was used because ≥15 years of age is recognised as being inclusive of Canada’s participating labour force population in Statistics Canada’s nationwide Labour Force Survey^(55)^.

## References

[1] Walker J , Lovett R , Kukutai T et al. (2017) Indigenous health data and the path to healing. Lancet 390, 2022–2023. 29115232 10.1016/S0140-6736(17)32755-1

[2] Reading CL & Wien F (2009) Health Inequalities and the Social Determinants of Aboriginal Peoples’ Health. Prince George, BC: National Collaborating Centre for Aboriginal Health.

[3] Richmond CA & Ross NA (2009) The determinants of first nation and Inuit health: a critical population health approach. Health Place 15, 403–411. 18760954 10.1016/j.healthplace.2008.07.004

[4] Adelson N (2005) The embodiment of inequity: health disparities in Aboriginal Canada. Can J Public Health 96, S45–S61. 10.1007/BF03403702 PMC6975716 16078555

[5] McIntyre L (2003) Food security: more than a determinant of health. Policy Opt 24, 46–51.

[6] Tarasuk V (2005) Household food insecurity in Canada. Top Clin Nutr 20, 299–312.

[7] Delormier T , Horn‐Miller K , McComber AM et al. (2017) Reclaiming food security in the Mohawk community of Kahnawake through Haudenosaunee responsibilities. Maternal Child Nutr 13, e12556. 10.1111/mcn.12556 PMC6865881 29359439

[8] Anderson I , Robson B , Connolly M et al. (2016) Indigenous and tribal peoples’ health (The Lancet–Lowitja Institute Global Collaboration): a population study. Lancet 388, 131–157. 27108232 10.1016/S0140-6736(16)00345-7

[9] Lemke S & Delormier T (2017) Indigenous peoples’ food systems, nutrition, and gender: conceptual and methodological considerations. Matern Child Nutr 13, e12499. 10.1111/mcn.12499 PMC6866027 29359433

[10] Statistics Canada (2017) Aboriginal Peoples in Canada: Key results from the 2016 Census. http://www.statcan.gc.ca/daily-quotidien/171025/dq171025a-eng.pdf (accessed November 2017).

[11] Statistics Canada (2013) Aboriginal Peoples in Canada: First Nations People, Métis and Inuit. Ottawa, ON: Government of Canada. Catalogue no. 99–011-X2011001. http://www12.statcan.gc.ca/nhs-enm/2011/as-sa/99–011-x/99–011-x2011001-eng.pdf (accessed November 2017).

[12] Egeland GM , Johnson-Down L , Cao ZR et al. (2011) Food insecurity and nutrition transition combine to affect nutrient intakes in Canadian Arctic Communities, 2. J Nutr 141, 1746–1753. 21753059 10.3945/jn.111.139006

[13] Kuhnlein HV , Receveur O , Soueida R et al. (2004) Arctic indigenous peoples experience the nutrition transition with changing dietary patterns and obesity. J Nutr 134, 1447–1453. 15173410 10.1093/jn/134.6.1447

[14] Gracey M & King M (2009) Indigenous health part 1: determinants and disease patterns. Lancet 374, 65–75. 19577695 10.1016/S0140-6736(09)60914-4

[15] Rotenberg CR (2016) Social Determinants of Health for the Off-reserve First Nations Population, 15 Years of Age and Older, 2012. Statistics Canada; Catalogue no. 89‐653‐X2016009. http://www.statcan.gc.ca/pub/89–653-x/89–653-x2016010-eng.pdf.

[16] Young TK , Reading J & Elias B (2000) Type 2 diabetes mellitus in Canada’s First Nations: status of an epidemic in progress. Can Med Assoc J 16, 561–566. PMC80466 11006768

[17] Haman F , Fontaine-Bisson B , Batal M et al. (2010) Obesity and type 2 diabetes in Northern Canada’s remote First Nations communities: the dietary dilemma. Int J Obesity 34, S24. 10.1038/ijo.2010.236 21151143

[18] Cancer Care Ontario (2016) Path to Prevention – Recommendations for Reducing Chronic Disease in First Nations, Inuit and Métis. Toronto: Queen’s Printer for Ontario.

[19] Public Health Agency of Canada (2011) Diabetes in Canada: Facts and Figures from a Public Health Perspective. Ottawa: Public Health Agency of Canada.

[20] Public Health Agency of Canada (2011) Obesity in Canada. Public Health Agency of Canada and Canadian Institute for Health Information. Ottawa: Public Health Agency of Canada.

[21] Gionet L & Roshanafshar S (2013) Select Health Indicators of First Nations People Living off Reserve, Métis and Inuit. Ottawa, ON: Statistics Canada.

[22] Kolahdooz F , Sadeghirad B , Corriveau A et al. (2017) Prevalence of overweight and obesity among indigenous populations in Canada: a systematic review and meta-analysis. Crit Rev Food Sci Nutr 57, 1316–1327. 26566086 10.1080/10408398.2014.913003

[23] Batal M & Decelles S (2019) A scoping review of obesity among indigenous peoples in Canada. J Obes 2019, 9741090. 31281674 10.1155/2019/9741090 PMC6589240

[24] Skinner K , Hanning RM & Tsuji LJ (2014) Prevalence and severity of household food insecurity of First Nations people living in an on-reserve, sub-Arctic community within the Mushkegowuk territory. Public Health Nutr 17, 31–39. 23806766 10.1017/S1368980013001705 PMC10282343

[25] Fieldhouse P & Thompson S (2012) Tackling food security issues in indigenous communities in Canada: the Manitoba experience. Nutr Diet 69, 217–221.

[26] Power EM (2008) Conceptualizing food security for Aboriginal people in Canada. Can J Public Health 99, 95–97. 18457280 10.1007/BF03405452 PMC6975848

[27] Daschuk JW (2013) Clearing the Plains: Disease, Politics of Starvation, and the Loss of Aboriginal Life, Vol. 65. Regina: University of Regina Press.

[28] Owen J (2019) Food as a Weapon in the Residential School System. Food Secure Canada. https://foodsecurecanada.org/residential-schools-and-using-food-weapon (accessed December 2019).

[29] Reeds J , Mansuri S , Mamakeesick M et al. (2016) Dietary patterns and type 2 diabetes mellitus in a First Nations community. Can J Diab 40, 304–310. 10.1016/j.jcjd.2016.05.001 27374251

[30] Batal M , Gray-Donald K , Kuhnlein HV et al. (2005) Estimation of traditional food intake in indigenous communities in Denendeh and the Yukon. Int J Circumpolar Health 64, 46–54. 15776992 10.3402/ijch.v64i1.17953

[31] Sheikh N , Egeland GM , Johnson-Down L et al. (2011) Changing dietary patterns and body mass index over time in Canadian Inuit communities. Int J Circumpolar Health 70, 511–519. 22152598 10.3402/ijch.v70i5.17863

[32] Gittelsohn J , Wolever TM , Harris SB et al. (1998) Specific patterns of food consumption and preparation are associated with diabetes and obesity in a Native Canadian community. J Nut 128, 541–547. 10.1093/jn/128.3.541 9482761

[33] Sharma S , Gittelsohn J , Rosol R et al. (2010) Addressing the public health burden caused by the nutrition transition through the healthy foods north nutrition and lifestyle intervention programme. J Human Nutr Diet 23, 120–127. 10.1111/j.1365-277X.2010.01107.x 21158971

[34] Tarasuk V , Mitchell A & Dachner N (2014) Household Food Insecurity in Canada, 2012. Research to Identify Policy Options to Reduce Food Insecurity. http://nutritionalsciences.lamp.utoronto.ca (accessed November 2017).

[35] First Nations Information Governance Centre (FNIGC) (2018) National Report of the First Nations Regional Health Survey Phase 3, Vol. 2. Ottawa, ON: First Nations Information Governance Centre (FNIGC).

[36] Huet C , Rosol R & Egeland GM (2012) The prevalence of food insecurity is high and the diet quality poor in Inuit communities – 3. J Nutr 142, 541–547. 22323760 10.3945/jn.111.149278

[37] Holben DH , Barnett MA & Holcomb JP (2007) Food insecurity is associated with health status of older adults participating in the commodity supplemental food program in a rural Appalachian Ohio county. J Hunger Environ Nutr 1, 89–99.

[38] Morales ME & Berkowitz SA (2016) The relationship between food insecurity, dietary patterns, and obesity. Curr Nutr Rep 5, 54–60. 29955440 10.1007/s13668-016-0153-y PMC6019322

[39] Olson CM (1999) Nutrition and health outcomes associated with food insecurity and hunger. J Nutr 129, 521S–524S. 10064322 10.1093/jn/129.2.521S

[40] Hanson KL , Sobal J & Frongillo EA (2007) Gender and marital status clarify associations between food insecurity and body weight. J Nutr 137, 1460–1465. 17513407 10.1093/jn/137.6.1460

[41] Wilde PE & Peterman JN (2006) Individual weight change is associated with household food security status. J Nutr 136, 1395–1400. 16614436 10.1093/jn/136.5.1395

[42] Townsend MS , Peerson J , Love B et al. (2001) Food insecurity is positively related to overweight in women. J Nutr 131, 1738–1745. 11385061 10.1093/jn/131.6.1738

[43] Adams EJ , Grummer-Strawn L & Chavez G (2003) Food insecurity is associated with increased risk of obesity in California women. J Nutr 133, 1070–1074. 12672921 10.1093/jn/133.4.1070

[44] Lyons AA , Park J & Nelson CH (2008) Food insecurity and obesity: a comparison of self-reported and measured height and weight. Am J Public Health 98, 751–757. 17666697 10.2105/AJPH.2006.093211 PMC2377006

[45] Stuff JE , Casey PH , Connell CL et al. (2007) Household food insecurity and obesity, chronic disease, and chronic disease risk factors. J Hunger Environ Nutr 1, 43–62.

[46] Drewnowski A & Specter SE (2004) Poverty and obesity: the role of energy density and energy costs. Am J Clin Nutr 79, 6–16. 14684391 10.1093/ajcn/79.1.6

[47] Domingo A (2016) Household food insecurity and obesity in First Nations communities in Canada. Thesis, University of British Columbia. 10.1017/S1368980019004889 PMC8025097 32366338

[48] Chan L , Receveur O , Batal M et al. (2016) First Nations Food, Nutrition and Environment Study (FNFNES): Results from Alberta, 2013. Ottawa, ON: University of Ottawa.

[49] Chan L , Receveur O , Batal M et al. (2014) First Nations Food, Nutrition and Environment Study (FNFNES): Results from Ontario (2011/2012). Ottawa, ON: University of Ottawa.

[50] Chan L , Receveur O , Sharp D et al. (2012) First Nations Food, Nutrition and Environment Study (FNFNES): Results from Manitoba (2010). Prince George, BC: University of Northern British Columbia.

[51] Chan L , Receveur O , Sharp D et al. (2012) First Nations Food, Nutrition and Environment Study (FNFNES): Results from British Columbia (2008/2009). Prince George, BC: University of Northern British Columbia.

[52] Canadian Institutes of Health Research, Natural Sciences and Engineering Research Council of Canada & Social Sciences and Humanities Research Council of Canada (2010) Tri-Council Policy Statement: Ethical Conduct for Research Involving Humans. Ottawa, ON: Interagency Secretariat on Research Ethics.

[53] First Nations Information Governance Centre (2007) OCAP: Ownership, Control, Access and Possession. Sanctioned by the First Nations Information Governance Committee, Assembly of First Nations. Ottawa: National Aboriginal Health Organization. Report published April 2007.

[54] Kuhnlein HV , Receveur O & Chan HM (2001) Traditional food systems research with Canadian indigenous peoples. Int J Circumpolar Health 60, 112–122. 11507960

[55] Health Canada (2013) Canadian Guidelines for Body Weight Classification in Adults. Ottawa, ON: Health Canada. Catalogue No. H49–179/2003–1E. https://www.canada.ca/content/dam/hc-sc/migration/hc-sc/fn-an/alt_formats/hpfb-dgpsa/pdf/nutrition/cg_quick_ref-ldc_rapide_ref-eng.pdf (accessed December 2017).

[56] Bush M & General RD (2007) Canadian Community Health Survey, Cycle 2.2, Nutrition (2004): Income-Related Household Food Security in Canada. Ottawa, ON: Office of Nutrition Policy and Promotion, Health Canada.

[57] Basiotis PP & Lino M (2003) Food insufficiency and prevalence of overweight among adult women. Family Econ Nutr Rev 15, 55.

[58] Willows ND , Veugelers P , Raine K et al. (2009) Prevalence and sociodemographic risk factors related to household food security in Aboriginal peoples in Canada. Public Health Nutr 12, 1150–1156. 19105863 10.1017/S1368980008004345

[59] Thompson S , Gulrukh A , Ballard M et al. (2011) Is community economic development putting healthy food on the table? Food sovereignty in Northern Manitoba’s Aboriginal communities. J Aboriginal Econ Dev 7, 14–39.

[60] Gionet L & Roshanafshar S (2013) Select Health Indicators of First Nations People Living Off Reserve, Métis and Inuit. Ottawa, ON: Statistics Canada.

[61] Guo Y , Berrang-Ford L , Ford J et al. (2015) Seasonal prevalence and determinants of food insecurity in Iqaluit, Nunavut. Int J Circumpolar Health 74, 27284. 26248959 10.3402/ijch.v74.27284 PMC4528079

[62] Kuhnlein HV , Burlingame B & Erasmus B (2013) Policy and strategies to improve nutrition and health for indigenous peoples. Indigenous peoples’ food systems and well-being: interventions and policies for healthy communities. Matern Child Nutr 13, 279–295.

[63] McIntyre L (2003) Food security: more than a determinant of health. Policy Opt Montreal 24, 46–51.

[64] Beaumier MC & Ford JD (2010) Food insecurity among Inuit women exacerbated by socioeconomic stresses and climate change. Can J Public Health 101, 196–201. 20737808 10.1007/BF03404373 PMC6973768

[65] Martin MA & Lippert AM (2012) Feeding her children, but risking her health: the intersection of gender, household food insecurity and obesity. Soc Sci Med 74, 1754–1764. 22245381 10.1016/j.socscimed.2011.11.013 PMC3338899

[66] Tarasuk V (2005) Household food insecurity in Canada. Top Clin Nutr 20, 299–312.

[67] Li N , Dachner N , Tarasuk V et al. (2016) Priority Health Equity Indicators for British Columbia: Household Food Insecurity Indicator Executive Summary. Vancouver, BC: Provincial Health Services Authority.

[68] Statistics Canada (2018) Labour Force Survey, June 2018. https://www150.statcan.gc.ca/n1/en/daily-quotidien/180706/dq180706a-eng.pdf?st=GSopF-aY (accessed August 2018).

[69] Blanchet C & Rochette L (2008) Nutrition and Food Consumption Among the Inuit of Nunavik. Nunavik Inuit Health Survey 2004, Qanuippitaa? How Are We? Québec/Kuujjuaq: Institut national de santé publique du Québec (INSPQ)/Nunavik Regional Board of Health and Social Services (NRBHSS).

[70] Power EM (2005) Determinants of healthy eating among low-income Canadians. Can J Public Health 96, S37–S42.16042163

[71] Provincial Health Services Authority (2016) Food Costing in BC 2015. Vancouver, BC: Provincial Health Services Authority, Population and Public Health Program.

[72] Béland D & Daigneault PM (editors) (2015) Welfare Reform in Canada: Provincial Social Assistance in Comparative Perspective. Toronto, ON: University of Toronto Press.

[73] Mead E , Gittelsohn J , Kratzmann M et al. (2010) Impact of the changing food environment on dietary practices of an Inuit population in Arctic Canada. J Human Nutr Diet 23, 18–26. 21158958 10.1111/j.1365-277X.2010.01102.x

[74] Bhawra J , Cooke MJ , Hanning R et al. (2015) Community perspectives on food insecurity and obesity: focus groups with caregivers of Métis and off-reserve First Nations children. Int J Equity Health 14, 96. 26475264 10.1186/s12939-015-0232-5 PMC4609156

[75] Batal M , Johnson-Down L , Moubarac JC et al. (2018) Quantifying associations of the dietary share of ultra-processed foods with overall diet quality in First Nations peoples in the Canadian provinces of British Columbia, Alberta, Manitoba and Ontario. Public Health Nutr 21, 103–113. 28738909 10.1017/S1368980017001677 PMC10260810

[76] Batal M , Johnson-Down L , Moubarac JC et al. (2018) Sociodemographic associations of the dietary proportion of ultra-processed foods in First Nations peoples in the Canadian provinces of British Columbia, Manitoba, Alberta and Ontario. Int J Food Sci Nutr 69, 753–761. 29252033 10.1080/09637486.2017.1412405

[77] Warbrick I , Came H & Dickson A (2018) The shame of fat shaming in public health: moving past racism to embrace indigenous solutions. Public Health 176, 128–132. 30352699 10.1016/j.puhe.2018.08.013

[78] Desmarais AA & Wittman H (2014) Farmers, foodies and First Nations: getting to food sovereignty in Canada. J Peasant Stud 41, 1153–1173.

